# Perspectives for pharmaceutical screening at XFEL sources using the example of Lassa virus endonuclease

**DOI:** 10.1107/S2059798326009435

**Published:** 2026-09-25

**Authors:** Sven Falke, Patrick Y. A. Reinke, Daniel Rosenberg, Pontus Fischer, Jan Meyer, Marina Galchenkova, Alexandra Tolstikova, Valerio Mariani, Johanna M. Senst, Philipp Lewe, Susanne Witt, Armin Wagner, Henry N. Chapman, Mark Hunter, Sebastian Günther, Alke Meents

**Affiliations:** a Center for Free-Electron Laser Science CFEL Deutsches Elektronen-Synchrotron DESY Notkestrasse 85 22607Hamburg Germany; b SLAC National Accelerator Laboratory Menlo Park California USA; c Deutsches Elektronen-Synchrotron DESY Notkestrasse 85 22607Hamburg Germany; d University Medical Centre Hamburg Eppendorf UKE and Centre for Structural Systems Biology CSSB, c/o DESY, Notkestrasse 85, 22607Hamburg, Germany; e Diamond Light Source DidcotOX11 0DE United Kingdom; f Department of Physics University of Hamburg Luruper Chaussee 149 22761Hamburg Germany; g The Hamburg Centre for Ultrafast Imaging, Luruper Chaussee 149, 22761Hamburg, Germany; University of Regensburg, Germany

**Keywords:** serial femtosecond X-ray crystallography, viral cap-snatching endonuclease, compound screening, drug development, room-temperature diffraction data collection, XFELs

## Abstract

Emerging human pathogenic RNA viruses such as Lassa virus (LASV) and closely related viruses such as Hantavirus and Andes virus continue to be a major health threat globally. Treatment options for infections are severely limited. The widely conserved cap-snatching endonuclease of LASV (LASVendoN) is therefore used as an interesting drug target for our X-ray compound-screening experiments. Considering recent improvements in instrumentation and serial crystallography method development, different approaches may be used to facilitate pharmaceutical compound screening. Current-generation X-ray free-electron laser (XFEL) sources hold promise for obtaining higher quality diffraction data and achieving a high sample throughput using fixed-target serial femtosecond X-ray crystallography. We collected serial fixed-target X-ray diffraction data at Linac Coherent Light Source and at the synchrotron source PETRA III for comparison and solved the crystal structures of 2,4-dioxo-4-phenylbutanoic acid (DPBA) and baloxavir acid (BXA) binding to the active site of LASVendoN. These solved structures represent a reasonable starting point for drug development. In addition to room-temperature diffraction data, conventional single-crystal diffraction data were recorded at 100 K for comparison. To analyse the sample throughput in a compound-screening experiment at an XFEL, we further present data for the bacterial fosfomycin-resistance protein A with six ligands previously identified in a synchrotron screening experiment. In summary, the XFEL and synchrotron data-collection approaches have proven to be useful for compound screening with minor differences in achievable data quality and resolution. The individual experimental setup, detector and potentially the use of beam sweeping at XFELs at close-to-physiological temperatures combined with reasonable sample consumption and minimized radiation damage are considered to be advantageous for high-throughput drug development.

## Abstract 2

To facilitate structure-based drug development, XFEL sources are of paramount interest. Here, we analyse the feasibility and data quality of serial femtosecond X-ray crystallography compound screening at Linac Coherent Light Source and compare it with PETRA III synchrotron experiments. For one Lassa virus model protein, we determined new crystal structures with two inhibitors.

## 1. Introduction

Emerging pathogens pose a major global health threat. Structure-assisted drug design is a very powerful method for developing new drugs and active compounds, which has gained additional significance in recent years through the use of AI-based methods (Zhang *et al.*, 2025 ▸) and higher throughput (Fearon *et al.*, 2025 ▸). Based on experimentally determined structural data that show how a molecule binds to a target protein, new molecules with improved properties, such as higher binding affinity and selectivity, better bio­availability or fewer side effects, are developed. Accurate and unambiguous knowledge of the binding pose is thereby essential for the success of the method. X-ray crystallography is currently the most powerful method for such required high-resolution structural investigations, as it allows the determination of many structures in a very short time. Recent improvements in instrumentation, the brightness of radiation sources, the availability of compound libraries as well as the automatization of crystal handling and diffraction data processing have helped to increase the throughput and made X-ray crystallographic screening widely accessible (Wollenhaupt *et al.*, 2020 ▸; Günther *et al.*, 2021 ▸). In such screening experiments, the achievable resolution is of high general importance for accurately determining binding poses and distances.

The vast majority of X-ray screening experiments are currently conducted at cryogenic temperatures of around 100 K or even lower. This approach makes it possible to protect the protein crystals from drying out during the investigation and thus losing their diffraction properties, and to keep radiation damage to a minimum. However, such temperatures are far away from relevant physiological temperatures above 273 K. Indeed, recent data indicate different ligand binding at cryogenic temperatures and physiological temperatures (Skaist Mehlman *et al.*, 2023 ▸; Günther *et al.*, 2025 ▸; Stachowski & Fischer, 2026 ▸).

X-ray structure determinations at room temperature prove to be significantly more challenging than conventional investigations at cryogenic temperatures, as only about 1/100 of the dose used for cryogenically cooled crystals can be applied due to the considerably greater radiation-sensitivity (Roedig *et al.*, 2016 ▸; de la Mora *et al.*, 2020 ▸). In conventional X-ray crystallography with single crystals, this usually leads to a significantly reduced resolution, which is a considerable limitation, especially for pharmaceutical screening experiments. In addition to global radiation damage, there is site-specific radiation damage including the reduction of metal ions and the formation of a variety of radicals, for example by the decarboxylation of glutamate or the breakage of disulfide bonds (Holton, 2009 ▸).

The method of serial crystallography (Martin-Garcia *et al.*, 2016 ▸; Günther *et al.*, 2025 ▸), which has become increasingly widespread in recent years, offers a remedy here. In contrast to conventional single-crystal structure analysis, which is based on the examination of ideally one or a few comparably large single crystals to acquire high-quality diffraction data, data sets from hundreds to thousands of single crystals are recorded and then combined into a complete data set. Because the absorbed photon energy is distributed among all of the crystals examined in the serial crystallography method, measurements can be performed at a significantly lower dose, thus achieving a significantly higher resolution in the experiments, especially at room temperature. In the first large-scale room-temperature fragment-screening experiment at PETRA III (Günther *et al.*, 2025 ▸) serial data collection gave higher resolution than conventional rotational experiments.

Ideally, the resolution achievable in serial crystallography experiments should be further increased using the method of serial femtosecond X-ray crystallography (SFX) at free-electron lasers (XFELs). In contrast to serial crystallography experiments at synchrotrons, where a typical dose limit applies, experiments at XFELs using extremely short femtosecond X-ray pulses allow significantly higher doses to be applied due to the ‘diffraction before destruction’ approach (Chapman *et al.*, 2014 ▸), usually resulting in even lower radiation-damage effects in the diffraction data. In addition, the significantly higher tolerable dose at XFELs should allow experiments to be carried out with significantly less sample material compared with synchrotrons. However, it should be noted that access to XFELs is scarce and the measurements are generally linked to high investment and operating costs of XFELs.

The data presented in this manuscript originate from a pharmaceutical X-ray screening campaign conducted at the MFX instrument at LCLS in October 2024, where we investigated several target proteins. These experiments aimed at exploring the potential of SFX for structure-based drug discovery and in particular whether ligand identification can be improved compared with serial crystallography experiments at synchrotrons (SSX; Günther *et al.*, 2025 ▸). We also compared the SFX data with conventional rotation data from cryogenically cooled samples. Previous studies investigated the potential benefits of SFX data collection over conventional X-ray data collection, for example for a radiation-sensitive metalloprotein (Moreno-Chicano *et al.*, 2022 ▸). In another study Lyubimov and coworkers concluded that the statistics of their XFEL diffraction data (CC_1/2_, *R*_work_ and *R*_free_) for the synaptotagmin-1–SNARE complex differed from those obtained for synchrotron data from similar crystals at cryogenic temperature, with a higher maximum resolution obtained at the XFEL (Lyubimov *et al.*, 2016 ▸). The authors also state that more state-of the-art experiments in this direction are desirable. However, important aspects for high-throughput screening experiments such as the efficient use of sample and beamtime and reliable sample delivery have not been considered.

In this manuscript, we focus on novel structures obtained for the Lassa virus cap-snatching endonuclease domain (LASVendoN) in complex with two inhibitors. LASVendoN is a highly relevant target for upcoming screening campaigns. Crystals of LASVendoN, which is located at the N-terminus of the Lassa virus L protein, were used. The entire L protein is a multi-functional protein with a molecular weight of approximately 250 kDa, which has been investigated by small-angle X-ray scattering and cryoEM and is essential for the replication of the virus (Vogel *et al.*, 2019 ▸; Kouba *et al.*, 2021 ▸)

Lassa virus is the causative agent of severe haemorrhagic fever and is endemic in wide areas of western Africa (Buckley *et al.*, 1970 ▸). Like other segmented, negative-strand RNA viruses, including Hantavirus and Andes virus, it belongs to the class *Bunyaviricetes*. For many viruses of this class, treatment options are still severely limited, despite several of the viruses being clearly prioritized by the World Health Organization for epidemic and pandemic preparedness (https://www.who.int/publications/m/item/pathogens-prioritization-a-scientific-framework-for-epidemic-and-pandemic-research-preparedness). The endonuclease domain of Lassa virus has been described as a highly attractive target protein for the development of antiviral drugs interfering with the propagation of the Lassa virus or even multiple closely related viruses (Ter Horst *et al.*, 2019 ▸; Wallat *et al.*, 2014 ▸). We determined its native structure and resolved the interaction of the endo­nuclease with two different derivatives of diketo carboxylic acid binding to the active-site metal ions.

To further evaluate the achievable throughput of our method, we present SFX data for fosfomycin-resistance protein A from *Klebsiella pneumoniae* (FosAKP) in complex with six previously identified ligands. FosAKP is a drug target, which provides resistance to the broad-spectrum antibiotic fosfomycin (Klontz *et al.*, 2017 ▸). The obtained SFX datasets complement recent synchrotron screening experiments (Günther *et al.*, 2025 ▸).

## 2. Materials and methods

### 2.1. Purification of LASVendoN

A fragment of the gene encoding the N-terminal amino acids 1–200 of the LASV L protein was fused with an N-terminal cleavable octahistidine-SUMO tag and cloned into pET-24a. The complete plasmid was obtained from GenScript, USA. *Escherichia coli* (DE3) Lemo21 cells were transformed in preparation for gene overexpression. LB medium was supplemented with kanamycin (50 µg ml^−1^), chloramphenicol (34 µg ml^−1^) and l-rhamnose (0.5 µ*M* ml^−1^). The cells were grown at 37°C under vigorous shaking. At an OD_600_ of 0.6 the temperature was reduced to 16°C and a final concentration of 0.4 m*M* isopropyl β-d-1-thiogalactopyranoside was added. After 16 h the cells were harvested by centrifugation at 5000*g* and stored at −80°C until further use. Cells were resuspended in 100 m*M* Tris–HCl pH 8.0, 500 m*M* NaCl, 10 m*M* imidazole–HCl pH 8.0, 5%(*v*/*v*) glycerol, 5 m*M* MgCl_2_, supplemented with DNaseI and lysozyme, and lysed using a microfluidizer. Cell debris was separated by centrifugation at 30 000*g* for 45 min and the cleared supernatant was loaded onto a HisTrap HP column (Cytiva, USA) for Ni–NTA affinity purification. After washing with a concentration of 50 m*M* imidazole, the target protein was eluted with 250 m*M* imidazole. The octahistidine tag was cleaved off using SUMO protease (for 16 h at 4°C; 1 mg protease per 20 mg target protein; in-house purification by the CSSB Protein Production Core Facility) and the protein solution was passed through a HisTrap HP column again to remove the protease and tag. Optimized purity and homogeneity were achieved by size-exclusion chromatography using a HiLoad 16/600 Superdex 75 pg column (Cytiva, USA) pre-equilibrated with 20 m*M* HEPES–NaOH pH 7.6, 150 m*M* NaCl, 2.5%(*v*/*v*) glycerol, 1 m*M* DTT, 5 m*M* MgCl_2_. The purity and molecular weight of LASVendoN were verified using SDS–PAGE and subsequent Coomassie staining.

### 2.2. Crystallization of LASVendoN

Initial LASVendoN crystals obtained using reservoir solution consisting of 24%(*v*/*v*) MPD, 5%(*v*/*v*) DMSO, 0.1 *M* MES pH 6.0, based on a condition reported by Reguera *et al.* (2016 ▸), had a disordered N-terminus that reached and partially occupied the active site, which is not favourable for compound screening. The crystals were therefore prepared differently. Purified LASVendoN was mixed with subtilisin A at a molar ratio of 1:700 and a final LASVendoN concentration of 7.5 mg ml^−1^ in 20 m*M* HEPES–NaOH pH 7.6, 150 m*M* NaCl, 5%(*v*/*v*) glycerol, 5 m*M* MgCl_2_. The solution was kept on ice and incubated for 75 min to allow limited proteolytic cleavage by subtilisin A. Subsequently, the protein solution was centrifuged for 5 min at 16 000*g* at 4°C and then mixed with an equal volume of crystallization solution [10%(*w*/*v*) PEG 10 000, 250 m*M* MgSO_4_, 100 m*M* Tris–HCl pH 9.0] to obtain rod-shaped crystals of >100 µm in length. This protocol is based on the conditions specified by Wallat *et al.* (2014 ▸). One round of microseeding was applied to optimize the homogeneity of the required crystal suspension. This crystallization protocol provided a molecular packing with one monomer per asymmetric unit and allowed residues 1–169 of the L-protein N-terminus to be resolved. The active site is well accessible and the solvent content is approximately 60%.

In preparation for data collection, compounds were dissolved in DMSO at a concentration of 20 m*M*. Compound solution was added slowly and stepwise to the obtained crystal suspension, resulting in diluted reservoir solution with a final concentration of 5%(*v*/*v*) DMSO and 1 m*M* compound. The same solution composition and soaking procedure was used for single-crystal rotational data collection.

### 2.3. Purification and crystallization of FosAKP

FosAKP was recombinantly produced in *E. coli* BL21(DE3)pLysS cells and purified by Ni–NTA affinity chromatography and size-exclusion chromatography as described previously (Klontz *et al.*, 2017 ▸; Günther *et al.*, 2025 ▸). FosAKP crystals were prepared and soaked for SFX experiments as described by Günther and coworkers for the SX screening of the F2X-Entry library (Günther *et al.*, 2025 ▸) and using the same sample holders. Briefly, FosAKP was crystallized on six-compartment Kapton chips (Section 2.4.1) directly. FosAKP at a concentration of 25 mg ml^−1^ in 75 m*M* NaCl, 10 m*M* HEPES pH 7.5 was supplemented with a final concentration of 6 m*M* MnCl_2_ and then mixed with an equal volume of 16%(*w*/*v*) PEG 3350, 0.25 *M* MgCl_2_, 0.2 *M* KBr, 0.1 *M* bis-Tris pH 5.5 and a 1/10 volume of a seed stock in 26%(*w*/*v*) PEG 3350, 0.25 *M* MgCl_2_, 0.2 *M* KBr, 0.1 *M* bis-Tris pH 5.5. The Kapton chip was then inserted into a 3D-printed crystal-growth chamber with a reservoir of 3 ml precipitant solution for vapour-diffusion crystallization and incubated at 20°C. The chips were removed from the crystal-growth chamber for application of fragments from the F2X-Entry library. Fragment solution (80 µl) at a concentration of 25 m*M* in 5%(*v*/*v*) DMSO was pipetted onto the crystals in the chip compartments. Namely, FosAKP crystals were soaked with compounds A6 (SMILES: CSc1nccnn1), A9 (SMILES: Nc1nsc2ncccc12), A12 (SMILES: CC(C)c1noc(O)n1), B2 (SMILES: C1Cc2ccccc2CN1), G8 (SMILES: CCNC(=O)CSc1nnc(o1)C(C)C) and G12 (SMILES: O=C(N\N=C\c1cccs1)C1CC1) from the F2X-Entry screen library (Wollenhaupt *et al.*, 2020 ▸). Sample holders were subsequently put back into the growth vessel and incubated for 24 h. Chips were prepared for data collection as described in Section 2.4.1.

### 2.4. SX data collection

Serial fixed-target data collection was conducted both at the MFX endstation at LCLS in Stanford, USA and at the HiPhaX instrument at the PETRA III synchrotron in Hamburg, Germany. For both experiments the Roadrunner goniometer, developed by the DESY-FS-BMX group and the company suna-precision GmbH, was used. The Roadrunner goniometer is specifically designed for fixed-target serial crystallography experiments from protein crystals with minimal sample consumption (Roedig *et al.*, 2017 ▸; Lieske *et al.*, 2019 ▸). It provides a measurement chamber with precisely controlled temperature and relative humidity for data collection. The Roadrunner has recently been employed for successful pharmaceutical screening experiments at room temperature with serial crystallography targeting the enzyme FosAKP (Günther *et al.*, 2025 ▸). Using the same hardware and in particular the same sample holders and sample-preparation techniques for both data-collection scenarios is beneficial for a direct comparison of the timeline and results from the two experiments, even though beam parameters vary.

#### 2.4.1. SFX (MFX, LCLS)

For serial data collection at the MFX endstation at LCLS a Roadrunner goniometer was installed at the beamline and fully implemented in the LCLS control system, which required extension of the *Janus* data-collection and control software of the Roadrunner by an EPICS interface (Meyer *et al.*, 2026 ▸).

**Figure 1:**
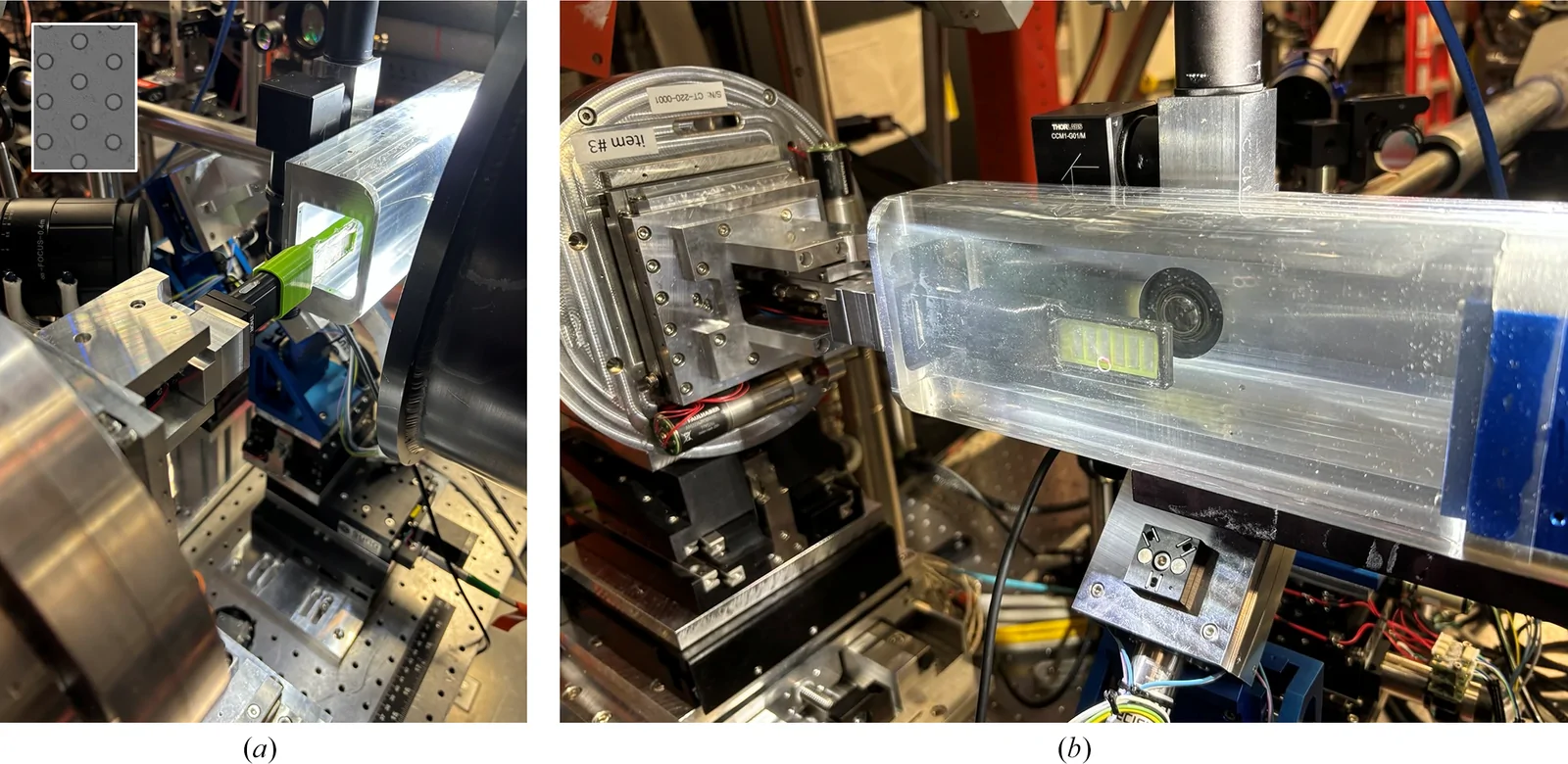
(*a*) Roadrunner goniometer for fixed-target SX, side view, in the mounting position with chip; the inset shows the Kapton foil of the chip with pores to facilitate blotting. (*b*) Roadrunner with a mounted chip in position for the experiment, front view, set up at MFX.

For sample presentation, Kapton chips with multiple compartments and equipped with a periodic pattern of micropores with a diameter of 10 µm (Fig. 1 ▸) were prepared by laser micro-machining. For the experiments, 80 µl LASVendoN crystal suspension was pipetted into each of the six compartments per chip and homogenously spread over the entire membrane area using a spatula. Excessive mother liquor was subsequently removed by blotting through the micropores. All sample-loading and handling steps were conducted in a glove box with a relative humidity of >96% to prevent the crystals from partially drying out to avoid non-isomorphism of the crystals, which would otherwise degrade the quality of the merged data. To prevent crystals from drying out during transport and loading onto the goniometer, the sample holders were protected with an X-ray-transparent cover maintaining a humid atmosphere for the crystals.

SFX data were recorded at an X-ray energy of 9.8 keV and the beam size at the sample position was around 3 µm at an X-ray pulse frequency of 120 Hz. For serial data collection the sample holders were raster-scanned through the X-ray beam. Fast scanning was performed in the horizontal direction with a spacing of 50 µm on the sample holder between two X-ray pulses. After completion of every line the chip was translated vertically by 36 µm and rotated by a small angular increment around the horizontal rotation axis of the goniometer before a new horizontal scan was started in the reverse direction. The scan ensured that measurements made in each compartment included a full a range of rotations from 0° to 20°. These images were subsequently grouped by compartment. This strategy increased the data-collection speed per chip. Diffraction patterns were recorded on an ePix10K-2.1M detector (van Driel *et al.*, 2020 ▸). Due to technical limitations of the gain-switching mode, the detector had to be operated in fixed low-gain mode for data collection. This resulted in a limited dynamic range of about 9000 X-ray photons per pulse and a loss of single photon-counting capability. We further had to attenuate the incoming X-ray beam to below 300 µJ pulse energy to avoid oversaturation and subsequent damage of the detector. These conditions resulted in an X-ray dose of typically about 15 MGy per exposure.

#### 2.4.2. SSX (HiPhaX, PETRA III)

Serial synchrotron data collection was conducted at the HiPhaX endstation at the PETRA III synchrotron. Data were collected with monochromatic radiation with an X-ray energy of 16 keV (0.7749 Å wavelength). The X-ray spot size at the sample position was approximately 20 × 20 µm with a photon flux of 3.5 × 10^11^ photons s^−1^. Diffraction data were recorded with a frame rate of 25 Hz on a PILATUS 6M detector. For data collection, a 12-compartment version of the chip was used with each compartment measuring 4.5 × 4.5 mm. Approximately 35 µl of LASVendoN crystal suspension was pipetted into one compartment. Scanning of the chips was performed compartment-wise. For scanning of each compartment, the sample holders were continuously translated in the horizontal direction at a speed of 0.375 mm s^−1^ during exposure to X-rays. With an exposure time of 40 ms per frame this led to an illuminated area of 15 × 20 µm (horizontal × vertical) per exposure and resulted in a dose of around 13 kGy. Again, after completion of every line, the chip was translated vertically and rotated slightly. In total about 40 000 images were recorded from every compartment, covering an angular rotation range of 0° to 30°.

### 2.5. Single-crystal data collection

Cryogenic X-ray diffraction data were collected at a temperature of 100 K from Kapton loop-mounted single crystals on beamline P11 at PETRA III using an X-ray energy of 12 keV (1.0332 Å wavelength) on an EIGER 2X 16M detector. The beam size was adjusted to 20 × 20 µm, with an attenuated photon flux of 4 × 10^12^ photons s^−1^. The average crystal size was about 150 × 20 × 20 µm, with 1800 images collected over 360° total rotation with 10 ms exposure per image. This resulted in an X-ray dose of about 50–76 MGy depending on the specific crystal shape and orientation.

### 2.6. Serial and single-crystal diffraction data processing

Data from the SFX experiments were pre-sorted using *OM* (*OnDA Monitor*) prior to further processing (Mariani *et al.*, 2016 ▸). SX data were processed using *CrystFEL* (v0.11.1; White *et al.*, 2012 ▸). The *peakfinder*8 algorithm was used to identify the Bragg peaks. Patterns were indexed using *XGANDALF* (Gevorkov *et al.*, 2019 ▸). Data were scaled and merged using *partialator* within *CrystFEL*. MTZ files for crystallographic data processing were generated from *CrystFEL* merged reflection data files using *F*2*MTZ* from the *CCP*4 program suite (Agirre *et al.*, 2023 ▸).

Single-crystal data were indexed and scaled using *XDS* (Kabsch, 2010 ▸). Initial reference coordinates were obtained by molecular replacement using *Phaser* (McCoy *et al.*, 2007 ▸) and PDB entry 4miw. All structures were further iteratively refined using established procedures in *phenix.refine* (Afonine *et al.*, 2012 ▸) and *Coot* (Emsley *et al.*, 2010 ▸). For all LASVendoN data sets, the maximum resolution was determined based on *I*/σ(*I*). Dataset statistics were calculated for a range of resolution cutoffs and the highest resolution was selected where *I*/σ(*I*) was still above 1. LASVendoN structure models and structure factors have been deposited in the PDB (https://www.rcsb.org/) with entry IDs 28jg, 28jh, 28ji, 28ua, 28ub, 28uc, 29ik, 29il and 29im. To reveal and compare changes in the datasets attributable to ligand binding, isomorphous *F*_o_(complex) − *F*_o_(apo) difference maps were calculated using *Xtrapol*8 with default parameters (De Zitter *et al.*, 2022 ▸). *F*_o_ − *F*_o_ maps are generally minimizing model bias.

## 3. Results

### 3.1. XFEL structures of LASVendoN and its complexes

Here, we determined the room-temperature structures of LASVendoN and its complexes with the inhibitors DPBA and BXA using fixed-target SFX at the MFX instrument at LCLS and using fixed-target SSX at the HiPhaX instrument at the PETRA III synchrotron. We further compare them with structures obtained with conventional single-crystal data collection at cryogenic temperatures collected on beamline P11, also located at PETRA III. Data-collection and refinement statistics from all three experiments are provided in Table 1 ▸.

**Figure 2:**
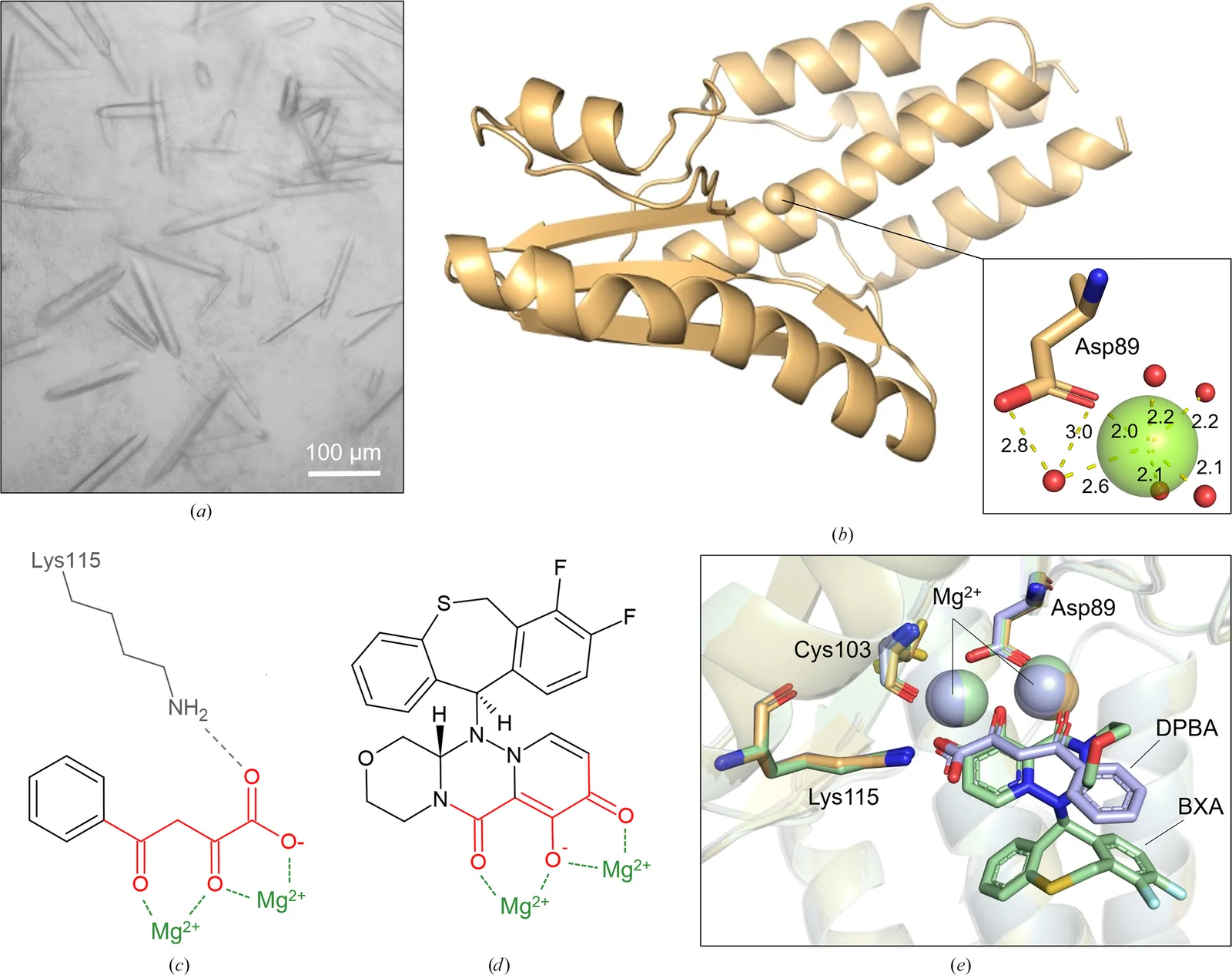
(*a*) LASVendo crystals used for data collection. (*b*) Cartoon representation of LASVendoN apo protein as solved by SFX. The inset shows the Mg^2+^ binding at the active site with distances shown in Å. (*c*) Structure and active-site interactions of 2,4-dioxo-4-phenylbutanoic acid (DPBA) and (*d*) baloxavir acid (BXA). The common diketo carboxylic acid scaffold is coloured red. (*e*) Superposition of the LASVendoN apo protein (orange), LASVendoN in complex with DPBA (light blue) and with BXA (green). Main-chain r.m.s.d. values of the protein–ligand complexes superimposed with the apo protein are below 1 Å.

Generally, this section describes the SFX structures, but the structures obtained with the different methods are in good agreement and no major structural differences were observed. LASVendoN is a monomer and has a high α-helix content, with three β-sheets interacting with each other in one stretch of the native protein (Fig. 1 ▸*a*). Despite individual loop regions, the protein is widely superimposable with cap-snatching endonucleases of related RNA viruses. Electron-density maps of the LASVendoN structures indicate very few occasional DMSO or short PEG molecules. The thiol group of Cys7 is prone to oxidation to cysteine sulfoxide and the Cys84 thiol group is either oxidized to cysteine sulfoxide or oxidized with covalently bound 1,4-dithiothreitol. Bivalent cations critically determine the catalytic activity of LASVendoN (Mg^2+^ > Mn^2+^ > Ca^2+^ > Zn^2+^), which mediate the binding and degradation of the RNA substrate (Wallat *et al.*, 2014 ▸). There is one bivalent magnesium ion that can clearly be located at the active site, coordinated by Asp89 and water molecules with octahedral geometry in the native apo structure (Figs. 2 ▸*b* and 2 ▸*e*). In the context of drug development, Mg^2+^ ions allow the active site to be targeted by a diketo carboxylic acid moiety via coordinating interactions with the cations. Screening of a few diketo carboxylic acid derivatives allowed us to identify the inter­action of LASVendoN with 2,4-dioxo-4-phenylbutanoic acid (DPBA) and baloxavir acid (BXA), as shown in Fig. 2 ▸.

In the presence of DPBA or BXA, the carbonyl O atoms of the diketo carboxylic acid scaffold put two Mg^2+^ ions from the solvent in a stable position. The position of one of the two Mg^2+^ ions remains nearly the same compared with the ion present in the apo structure. The free electron pairs of the carbonyl oxygen atoms of the diketo carboxylic acid (Figs. 2 ▸*c* and 2 ▸*d*; coloured red) interact with the cations, comparable to a Lewis acid–base interaction, and contribute to the octahedral coordination of the cations. Further, surrounding water molecules coordinating the Mg^2+^ ions gain order and the carboxy group of Asp89 and the backbone carbonyl oxygen of Cys103 also contribute to the octahedral coordination. As a consequence, the distance between the Mg^2+^ ions at the active site is 3.6 Å in both compound structures. While DPBA and BXA both share this Mg^2+^ coordination, the carboxy group of DPBA forms an additional 3.2 Å hydrogen bond with the ɛ amino group of Lys115. The phenyl ring of DPBA is solvent-exposed. The ‘bulky’ dibenzothiepin moiety of BXA does not interact with the active site as verified using *PoseEdit* (https://proteins.plus/).

### 3.2. Quality comparison of LASVendoN data

**Figure 3:**
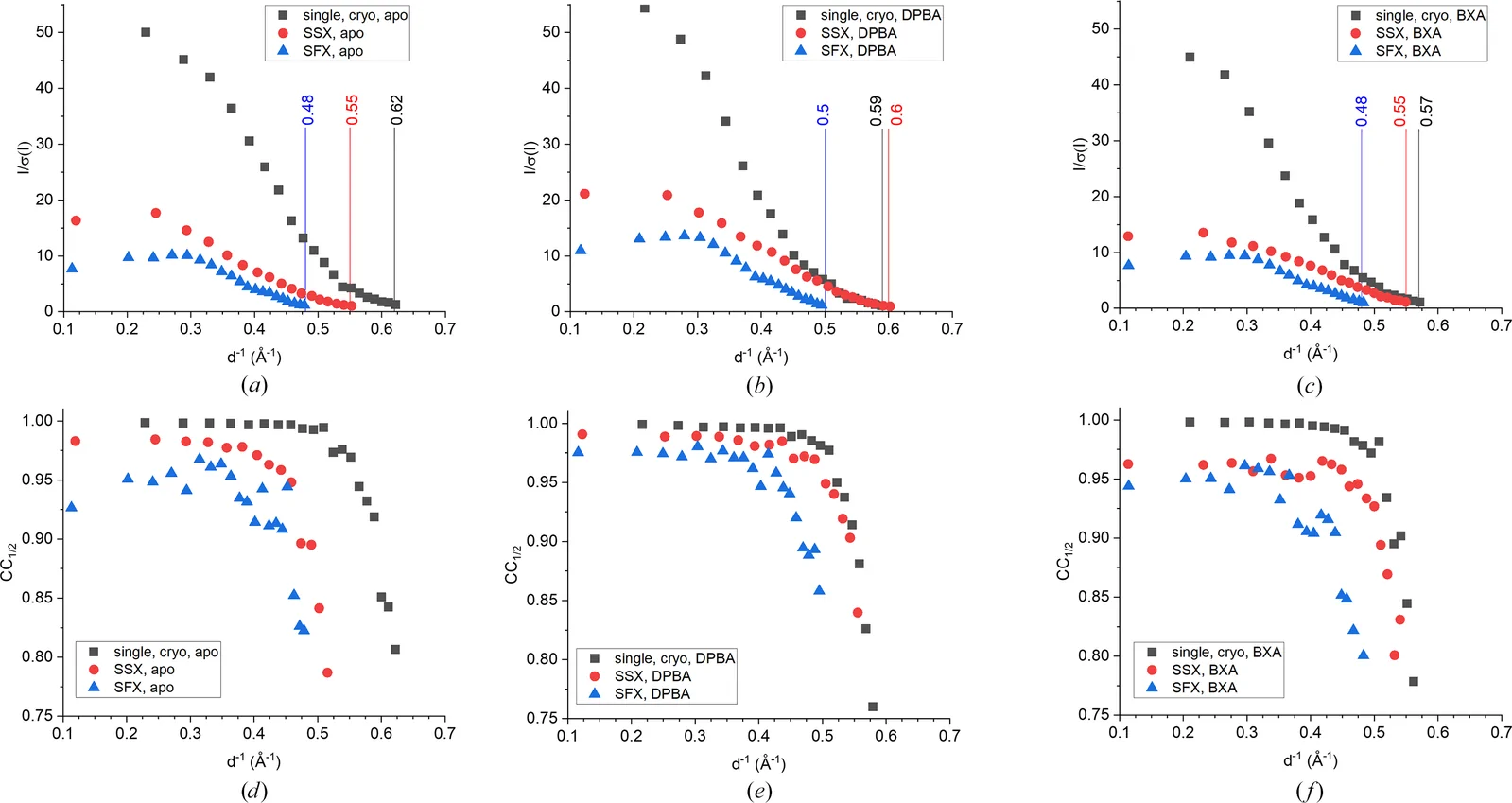
*I*/σ(*I*) and CC_1/2_ depending on the resolution shells of the diffraction data sets collected using SFX, SSX and single-crystal rotational crystallography: (*a*, *d*) LASVendoN apo data sets, (*b*, *e*) in complex with DPBA and (*c*, *f*) in complex with BXA. The maximum resolution was determined based on *I*/σ(*I*). The completeness in the outer resolution shell of all datasets is 100%. Further parameters are listed in Table 1 ▸.

Comparison of the data-processing parameters *I*/σ(*I*) and CC_1/2_ as a function of resolution for the apo structure and the two inhibitor complexes generally shows that cryogenic single-crystal data exhibit the strongest signal-to-noise ratios and show the highest correlation coefficients (Fig. 3 ▸). This is a typical observation when comparing single-crystal with multi-crystal data. Based on *I*/σ(*I*), the highest resolution data at 1.61 Å were obtained with single-crystal data collection from the apo form. SX data from the synchrotron yielded a resolution limit of 1.81 Å and SFX data yielded a resolution limit of 2.07 Å. For the two inhibitor structures no major differences between the single-crystal cryo data (1.7 and 1.75 Å for DPBA and BXA, respectively) and serial synchrotron data (1.66 and 1.82 Å) could be observed. In contrast, the SFX data were of lower resolution (2.02 and 2.07 Å). The same trend is observed for CC_1/2_. Comparison of the unit cells at room temperature determined with synchrotron SX and single-crystal cryogenic data shows an expected decrease in the unit-cell volume at cryogenic temperature by around 2–3%. The unit cells of the three structures determined with SFX are only slightly larger than the unit cells at cryogenic temperature (Table 1 ▸). The unit cell of the complex with DPBA has a shorter *c* axis and lower unit-cell volume, which is presumably caused by DPBA.

**Figure 4:**
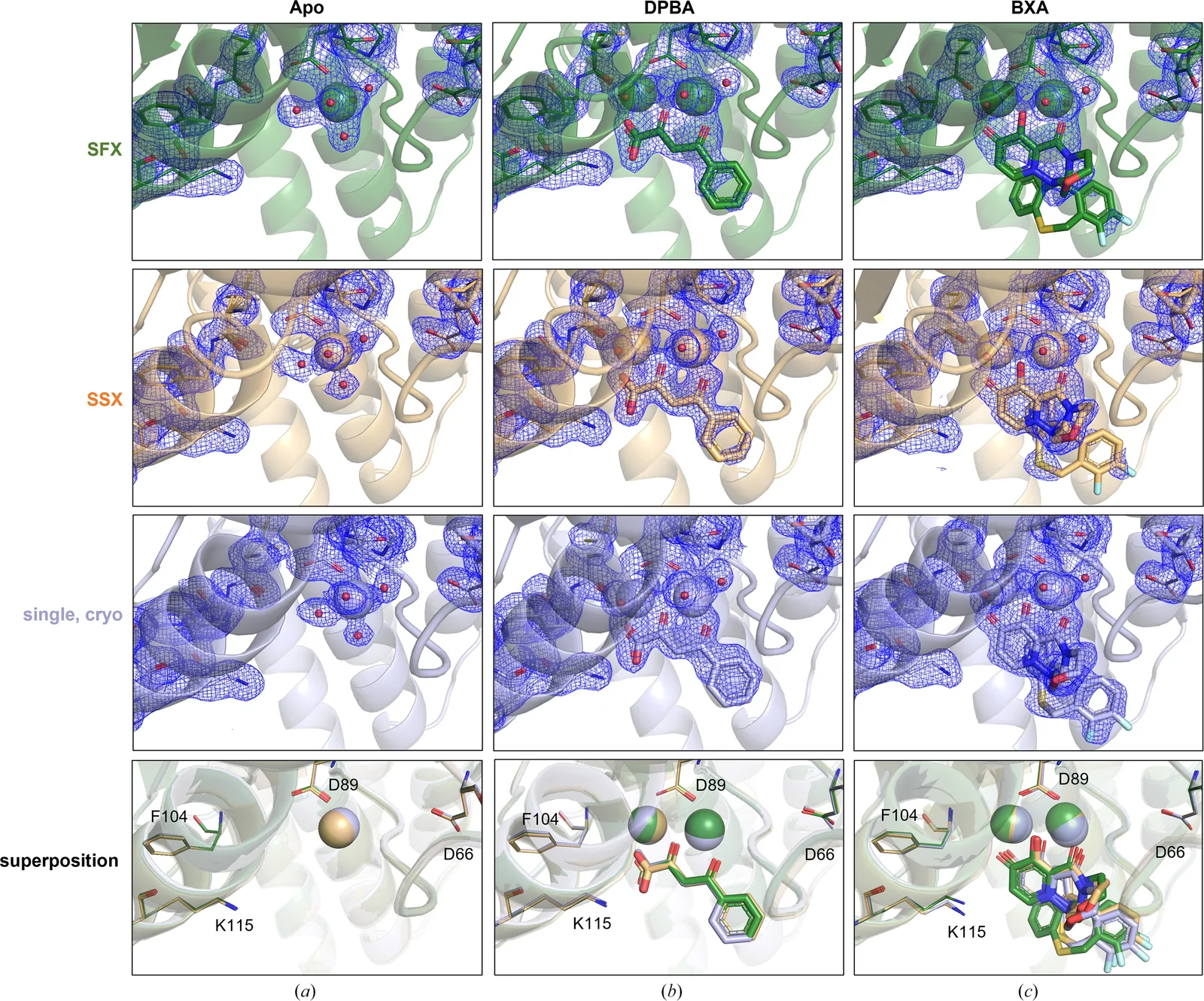
Comparison of active sites and ligand poses. 2*mF*_o_ − *DF*_c_ density maps of (*a*) the apo protein structures, (*b*) the DPBA complexes and (*c*) the BXA complexes. Maps are shown as blue mesh at a 1σ contour level.

The active-site 2*mF*_o_ − *DF*_c_ maps of the structures are provided in Fig. 4 ▸. The active sites of the determined apo protein structures do not show significant differences between the three different methods (Fig. 4 ▸*a*). The only difference observed is a sulfate ion close to Lys115, which can only be unambiguously modelled in the cryo-structure and likely originated from the crystallization solution. This observation is potentially caused by lower thermal motion at 100 K. Similarly, for the two inhibitor complexes no structural differences in the active-site residues are observed, and the ligand poses of DPBA and BXA are approximately identical. Only the electron density of the phenyl ring of DPBA is slightly higher in the dataset collected on beamline P11 at 100 K. The electron density of BXA in the structure determined at 100 K is well defined, including the dibenzothiepin moiety. In both structures collected using serial crystallo­graphy, the electron density of BXA is much lower at the dibenzothiepin moiety, indicating increased disorder at higher data-collection temperature. This moiety was also modelled in a different rotational position in the refined model of the SFX structure compared with the other two BXA structures. Further, there is a minor conformational difference of the morpholine ring of the BXA molecule in the BXA complex structures (Fig. 4 ▸*c*). An increased disorder of the dibenzo­thiepin moiety of BXA is also reflected by lower real-space CC (RSCC) values for BXA in the serial crystallography structures compared with rotational data collection at 100 K, indicating a better density-map fit at 100 K. The RSCC values for DPBA, in comparison, are nearly identical for the three methods and are >0.9. An estimate of the occupancy of both compounds based on extrapolated structure-factor amplitudes using *Xtrapol*8 indicates only minor differences between the three methods (Table 2 ▸).

**Figure 5:**
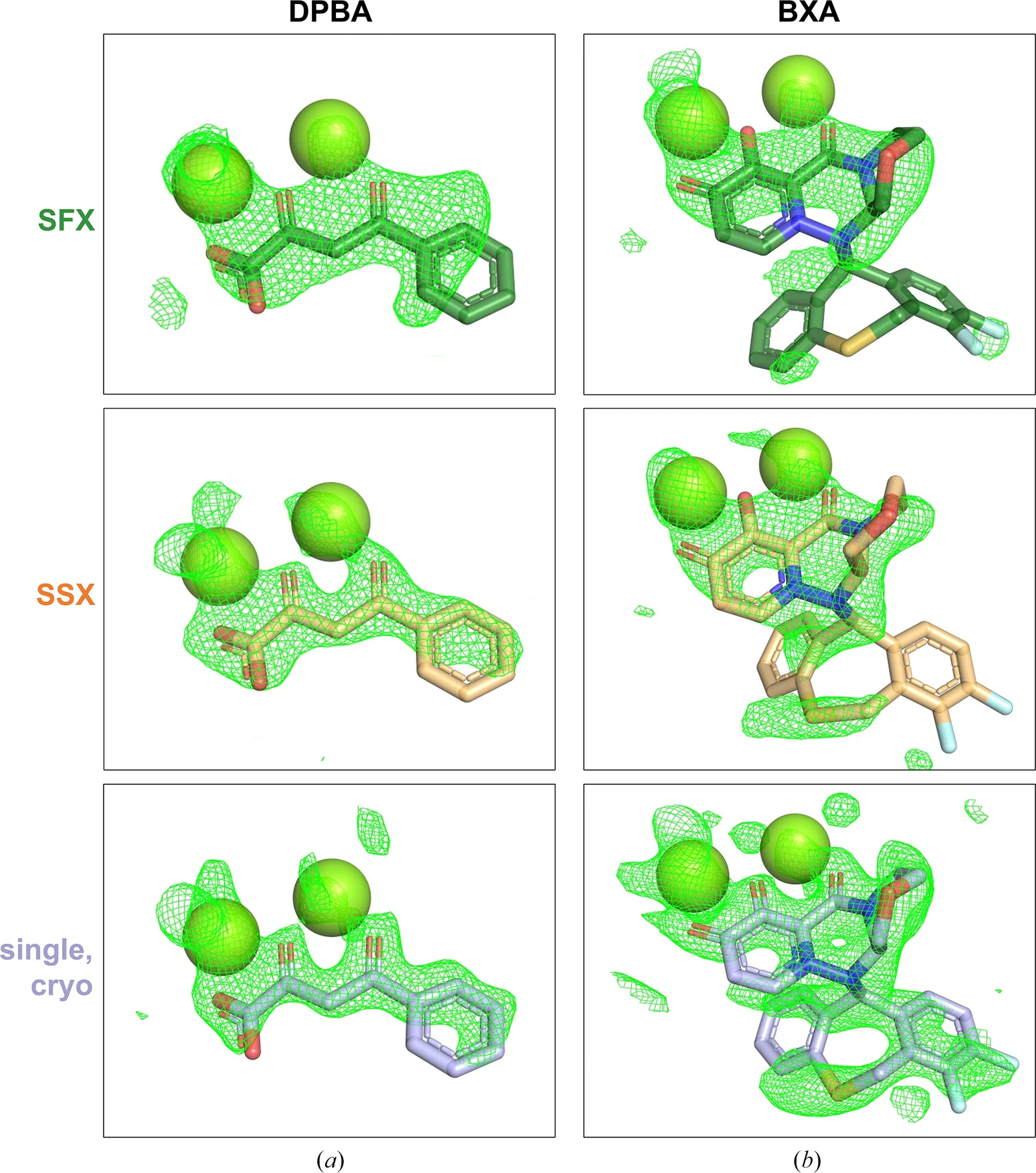
Isomorphous *F*_o_(complex) − *F*_o_(apo) maps defining the compound binding in the LASVendoN structures with (*a*) DPBA and (*b*) BXA. Maps are contoured at 3σ and shown within 3 Å of the compound and Mg^2+^ ions.

One common method of ligand identification in crystallo­graphy is the use of isomorphous *F*_o_ − *F*_o_ maps. In general, clear signal for both inhibitors is obtained by all three methods (Fig. 5 ▸). As already observed in the 2*mF*_o_ − *DF*_c_ density maps for the BXA complexes, single-crystal cryo data provide the strongest signal covering nearly the entire ligand. Similarly, there is strong signal for the entire DPBA ligand in the *F*_o_ − *F*_o_ maps, clearly in the map of the single-crystal cryo structure. The differences in compound peak height and size (measured as integral of density above 3.5σ around the peak) of DPBA and more obviously of BXA (Table 2 ▸) in the *F*_o_ − *F*_o_ maps is correlated with the data-collection temperature. The peak heights and volumes were calculated by *Xtrapol*8. The positive *F*_o_ − *F*_o_ density of the Mg^2+^ ions and coordinated water molecules is further evidence for stabilization of the active-site structure at the interface upon complex formation.

### 3.3. SFX fragment screening of FosAKP

In addition to LASVendoN data, datasets of FosAKP apo crystals and crystals soaked with one of six fragments from the F2X-Entry library were collected on the MFX instrument at LCLS. Data-collection times for screening of this subset of the library have been determined as shown in Supplementary Table S1. On average 43 950 diffraction pattern were recorded per compound, which required about 15 min of MFX beamtime for each of the seven FosAKP structures (Supplementary Table S1). Data-quality statistics of the seven FosAKP SFX datasets are provided in Supplementary Table S2. For comparison, statistics of SSX and single-crystal cryo datasets with the same compounds and using the same sample preparation are shown in Supplementary Tables S3 and S4, respectively, as adapted from Günther *et al.* (2025 ▸). We observed a slightly lower resolution and higher *B* factors for the SFX datasets in comparison to the synchrotron data, which is in line with the findings from the LASVendoN data.

## 4. Discussion and outlook

Each of the datasets collected with the three different diffraction modalities provides sufficient resolution for an unambiguous description of the binding interaction between two inhibitors and LASVendoN, forming the basis for downstream drug development.

### 4.1. Development of broad-spectrum diketo carboxylic acid inhibitors

As found here, diketo carboxylic acids allow the replacement of conserved water molecules and interaction with active-site bivalent cations of an enzyme drug target. Rational derivatization of these compounds has been used to target different enzymes and to counteract emerging drug resistance (Hazuda *et al.*, 2000 ▸; Noshi *et al.*, 2018 ▸; Ivashchenko *et al.*, 2021 ▸; Andreu *et al.*, 2025 ▸). A scaffold highly similar to typical diketo carboxylic acids such as BXA, DPBA or L-742,001 is provided by the three neighbouring O atoms of the natural compound thujaplicinol, which is found in cypress species and was shown to inhibit RNase H activity of Human immunodeficiency virus 1 by interacting with two active-site Mn^2+^ ions (Himmel *et al.*, 2009 ▸). A screen performed by Chen and coworkers revealed some inhibition of the influenza cap-snatching endonuclease by a 1,3,4,6-hexanetetrone scaffold (Chen *et al.*, 2013 ▸), which can be considered as an extended diketo carboxylic acid derivative with an additional carbonyl group.

X-ray screening of novel diketo carboxylic acid derivatives can aim at a higher specificity and affinity for our target LASVendoN. The structural conservation of endonuclease active sites of bunyaviruses presumably allows the targeting of multiple viruses and the maintainace of specificity over human endonucleases (Ter Horst *et al.*, 2019 ▸) to develop a broad-spectrum drug. This is facilitated by the availability of an increasing number of high-resolution Bunyavirus endo­nuclease structures in the PDB. Among others, endonuclease structures of Hantavirus, Andes virus and La Crosse virus have been solved (Reguera *et al.*, 2010 ▸, 2016 ▸; Fernández-García *et al.*, 2016 ▸; Feracci *et al.*, 2024 ▸). Notably, derivatives of BXA and L-742,001 have recently underlined their relevance for inhibiting a spectrum of RNA virus endonucleases. For instance, derivatives target the endonucleases of Ebinur Lake virus (Kuang *et al.*, 2022 ▸) and multiple nairoviruses (Kuang *et al.*, 2026 ▸) and target the pUL15 endonuclease of Herpes simplex virus 1, showing promising antiviral activity in mice (Andreu *et al.*, 2025 ▸). The approval to use baloxavir marboxil as an influenza drug, sold as Xofluza by Roche, has been extended and broadened in the European Union in 2023 and in the USA in 2022. In the future, further broad-spectrum drug development may be supported by artificial intelligence, allowing the integration of huge amounts of structural data and minimization of the side effects of drug candidates (Zhang *et al.*, 2025 ▸)*.*

### 4.2. Comparison of the data quality from different data-collection modalities

In summary, all three modalities used in this comparative study yielded high-quality structural data for the three examined LASVendoN sample systems. It should be emphasized that in addition to the established method of conventional cryo-crystallography, the method of fixed-target serial crystallography using the Roadrunner goniometer at room temperature has reached a level of maturity that allows this technique to be used very reliably and with low sample consumption both in the data collections with the Roadrunner goniometer at the MFX instrument at LCLS and at the HiPhaX P09 measurement station at the PETRA III synchrotron (Günther *et al.*, 2025 ▸). These measurements can be performed with a high degree of standardization and reliability. The method generally qualifies for routine use in pharmaceutical screening experiments at room temperature at both synchrotron-radiation sources and at XFELs, where, in particular at the latter, beamtime is severely limited.

In terms of data-quality parameters, the best data from two of the three LASVendoN sample systems examined were obtained using conventional cryogenic data collection at the synchrotron. The serial data from the synchrotron follow very closely in terms of resolution and CC_1/2_ values; in the case of the DPBA complex, the serial synchrotron data even exhibit the best resolution here. Overall, the values for the datasets obtained with the two methods are quite similar. Even in the electron-density maps only marginal differences are observed, perhaps apart from the visibility of a sulfate ion in one of the cryo datasets.

Somewhat unexpectedly, the LASVendoN data collected at the XFEL exhibit the lowest resolution for all three examined systems, and the other parameters also show slightly lower quality compared with the other modalities (Fig. 3 ▸, Table 1 ▸). This trend is confirmed by the FosAKP data. Differences in quality between the crystal preparations studied can largely be ruled out, as the sample preparation was carried out the same way. Nor can this be attributed to insufficient intensity of the incident X-rays, since by far the highest primary intensities were used for the XFEL experiments. In any case, among the different setups, a relatively significant limitation in the experiments could originate from the ePix detector, resulting from ‘gain switching’, which often causes scaling problems (Haubro *et al.*, 2025 ▸). To avoid discontinuities in the reflection intensities arising from gain switching and the resulting deterioration in data quality, we conducted our experiments at a fixed low-gain setting. In this mode, at a photon energy of 9.8 keV, the detector has a dynamic range of approximately 9000 photons which are read out using a 14-bit ADC, with a specified noise level of 2 analog-to-digital units (ADU; Blaj *et al.*, 2019 ▸). This results in a significantly reduced dynamic range and single-photon sensitivity is not achieved, which is particularly relevant here. For comparison, state-of-the-art photon-counting detectors such as the EIGER detectors (Dectris, Swiserland) provide a much larger dynamic range. The significantly lower dynamic range of the ePix detector should primarily affect the weaker reflections at high resolution, which could explain the observed differences in resolution and the higher Wilson *B* factors in the XFEL experiments.

### 4.3. Perspectives and methodology for pharmaceutical screening at FEL sources

Even though the resolution achieved at the XFEL in this case was slightly lower than that obtained at the synchrotron, pharmaceutical screening experiments at XFELs nonetheless provide great potential. The LASVendoN examined in this study is a relatively small protein of 20 kDa, from which we were able to grow sufficiently large, high-quality crystals for diffraction experiments, making it well suited for analysis at the synchrotron. At the same time, these crystals can benefit only minimally from the advantages of XFEL sources, such as the extremely high beam intensities combined with extremely small foci for the study of microcrystals and nanoparticles or by outrunning radiation-damage effects (Nass, 2019 ▸; Williams *et al.*, 2025 ▸). *In vivo* grown microcrystals (Redecke *et al.*, 2013 ▸) and membrane proteins such as G protein-coupled receptors (GPCR) have been studied at XFELs at high resolution (Johansson *et al.*, 2019 ▸; Kim *et al.*, 2023 ▸). For example, when Kim and coworkers solved the structures of the GPCR corticotropin-releasing hormone receptor 1 with selected antagonistic compounds by fixed-target SFX and Johansson and coworkers investigated human melatonin receptors, XFEL experiments have proven to be useful in drug development. The reduced radiation-damage effects provide unique opportunities for screening of protein targets where only microcrystals are available or for highly radiation-sensitive metalloenzymes. Examples of this include the copper nitrite reductase, the manganese-containing protein cluster of photosystem II, which releases oxygen from water molecules in photosynthesis, and heme cofactor-containing oxido­reductases such as cytochrome P450 (Suga *et al.*, 2020 ▸; Hough & Owen, 2021 ▸; Rose *et al.*, 2021 ▸; Nagao *et al.*, 2025 ▸).

A general limitation for pharmaceutical screening experiments at XFELs however stems from the fact that measurement time is both very expensive and scarce. Experiments may therefore be limited to challenging sample systems that can rather clearly benefit from the advantages of XFELs, allowing them to realize their full potential. In addition, the available measurement time should be used very efficiently. In the context of pharmaceutical screening, for example, an informed selection of the compounds that constitute the screen library is vital. A common fragment library such as the F2X-Universal screen (1103 compounds) consists of around 1000 compounds. The F2X-Entry screen, which is a part of the F2X-Universal screen, typically yields hit rates of more than 10% (Wollenhaupt *et al.*, 2020 ▸; Lennartz *et al.*, 2025 ▸; Günther *et al.*, 2025 ▸). With the current screening throughput at XFEL sources and the related costs it appears unrealistic to conduct a complete screen of this size. As suggested by Günther and coworkers, a preselection of hit compounds previously identified, for example in a conventional crystallographic cryo screen, can reduce the screening efforts to a reasonable size (Günther *et al.*, 2025 ▸).

However, the experimental efficiency strongly depends on the experimental setup. The Roadrunner goniometer used in both of our serial data-collection methods, along with the associated sample-preparation techniques and the use of multi-compartment Kapton chips, already meet these efficiency requirements perfectly. This allowed us to perform a total of 36 serial structure determinations in 9.6 h (82% of the shift time was used for data collection, excluding beamline setup at the beginning of the shift; number of recorded patterns, 1 397 579; hit rate, 0.62; indexing rate, 0.44), corresponding to a time of approximately 16 min per structure, including sample exchange and alignment, which accounts for approximately 3 min per structure; this total time is slightly lower than for the SSX data collection, however, it is still far away from the times of under 2 min per structure typically achievable with single-crystal measurements at synchrotron sources. In the present case, data collection was further limited by the LCLS’s 120 Hz pulse rate. Here, newer high-repetition-rate XFELs such as LCLS-II HE or the European XFEL, operating in the kilohertz and megahertz ranges, open up new possibilities for high-throughput screening experiments. Fast chip scanning has already been demonstrated for pulse rates up to 1000 Hz (Tolstikova *et al.*, 2019 ▸). With the newly developed beam-sweeping technique, even experiments with megahertz pulse rates become possible, which should require less than a minute per structure determination (Rodrigues *et al.*, in review).

In addition to the scanning hardware, such experiments also require the appropriate monitoring and analysis software to make efficient use of the measurement time. In our experiments, the *OM* program allowed us to receive near-real-time feedback on data quality and, when in doubt, to quickly adjust the measurement parameters (Mariani *et al.*, 2016 ▸). At higher repetition rates, real-time monitoring becomes significantly more challenging. Finally, the detectors must also support the appropriate data-acquisition frequencies and consistently deliver high data quality while maintaining a sufficient dynamic range. When all of these challenging needs are met, pharmaceutical screening experiments at XFELs could provide a highly valuable contribution to large-scale drug-development projects.

## Supplementary Material

PDB reference: Lassa virus cap-snatching endonuclease, apo, single crystal, cryo, 28ji

PDB reference: SFX, 29ik

PDB reference: SSX, 28ua

PDB reference: complex with 2,4-dioxo-4-phenylbutanoic acid, single crystal, cryo, 28jh

PDB reference: SFX, 29il

PDB reference: SSX, 28ub

PDB reference: complex with BXA, single crystal, cryo, 28jg

PDB reference: SFX, 29im

PDB reference: SSX, 28uc

Supplementary Tables. DOI: 10.1107/S2059798326009435/raj5003sup1.pdf

## Figures and Tables

**Table 1:** Data-collection parameters and refinement statistics for the LASVendoN SFX data sets obtained and complementary datasets collected at the PETRA III synchrotron Values in parentheses are for the outer shell.

Experiment/X-ray source	SFX (MFX, LCLS)			Serial synchrotron crystallography (HiPhaX, P09, PETRA III)			Rotational single crystal, cryo (P11, PETRA III)		
Compound	Apo, DMSO	DPBA	BXA	Apo, DMSO	DPBA	BXA	Apo, DMSO	DPBA	BXA
Data collection									
Wavelength (Å)	1.265	1.265	1.265	0.77490	0.77490	0.77490	1.03322	1.03322	1.03322
Space group	*P*4_3_2_1_2	*P*4_3_2_1_2	*P*4_3_2_1_2	*P*4_3_2_1_2	*P*4_3_2_1_2	*P*4_3_2_1_2	*P*4_3_2_1_2	*P*4_3_2_1_2	*P*4_3_2_1_2
*a* = *b*, *c* (Å)	57.70, 135.57	57.85, 133.85	57.73, 135.15	58.63, 135.64	58.37, 135.03	58.63, 135.57	57.69, 135.55	57.85, 133.86	57.72, 135.14
α = β = γ (°)	90	90	90	90	90	90	90	90	90
Unit-cell volume (Å^3^)	451352	447945	450422	466259	460054	466019	451128	447979	450232
Resolution (Å)	21.04–2.07 (2.12–2.07)	21.04–2.02 (2.05–2.02)	21.04–2.07 (2.10–2.07)	45.21–1.81 (1.86–1.81)	45.01–1.66 (1.69–1.66)	45.19–1.82 (1.85–1.82)	53.08–1.61 (1.64–1.61)	53.10–1.70 (1.73–1.70)	53.08–1.75 (1.78–1.75)
No. of patterns/No. of hits	83036/31659	124533/55291	124512/34851	40040/16431	40040/15415	40040/12021	—	—	—
Indexed patterns/indexed crystals	28841/40116	50741/69891	31390/41115	13994/21528	12924/21417	9215/15529	—	—	—
Total reflections	10962379 (465978)	22824145 (678442)	11709344 (377998)	2277052 (66773)	5742072 (64758)	2155081 (52322)	812943 (41064)	685010 (34173)	628807 (30760)
Unique reflections	14959 (940)	16532 (779)	15394 (762)	22831 (1736)	29030 (1404)	22818 (1118)	30633 (1501)	25943 (1251)	23901 (1136)
Multiplicity	732.8 (495.7)	1380.6 (870.9)	760.6 (496.1)	99.7 (38.5)	197.8 (46.1)	94.4 (46.8)	26.5 (27.4)	26.4 (27.3)	26.3 (27.1)
*R*_meas_ (%)	n.d.	n.d.	n.d.	n.d.	n.d.	n.d.	10.2 (351.7)	12.6 (427.5)	15.5 (497.0)
*R*_split_ (%)	13.95 (46.34)	10.39 (38.24)	14.64 (44.09)	10.87 (84.65)	8.39 (101.65)	12.86 (70.21)	n.d.	n.d.	n.d.
Mean(*I*)/σ(*I*)	8.5 (1.0)	7.3 (1.2)	5.3 (1.0)	7.0 (1.1)	8.3 (1.0)	6.4 (1.1)	16.9 (1.3)	15.9 (1.1)	14.1 (1.1)
CC*† (%)	99.2 (94.5)	99.6 (96.1)	99.2 (94.3)	99.7 (86.8)	99.8 (82.7)	99.5 (91.1)	99.9 (94.5)	99.9 (91.4)	99.9 (92.2)
CC_1/2_ (%)	96.7 (80.7)	98.4 (85.8)	96.8 (80.1)	98.9 (60.5)	99.4 (52)	97.8 (70.9)	99.9 (80.7)	99.9 (71.7)	99.9 (73.9)
Wilson *B* (Å^2)^	43.30	39.53	41.70	24.41	22.55	24.69	25.91	31.00	27.88
Completeness (%)	100 (100)	100 (100)	100 (100)	100 (100)	100 (100)	100 (100)	100 (100)	100 (100)	100 (100)
Refinement									
Resolution (Å)	20.598–2.070 (2.144–2.070)	20.598–2.020 (2.092–2.020)	20.598–2.070 (2.144–2.070)	41.458–1.810 (1.875–1.810)	44.156–1.660 (1.719–1.660)	44.344–1.820 (1.885–1.820)	53.080–1.608 (1.666–1.608)	53.101–1.698 (1.759–1.698)	53.083–1.749 (1.812–1.749)
Reflections in refinement	14869 (1451)	15958 (1547)	14869 (1449)	22363 (2169)	28388 (2757)	22003 (2153)	30574 (2985)	25614 (2527)	23890 (2318)
*R*_work_/*R*_free_	0.2047 (0.3414)/0.2342 (0.3875)	0.1903 (0.2389)/0.2305 (0.2855)	0.2006 (0.3927)/0.2352 (0.4078)	0.1822 (0.2988)/0.2164 (0.3029)	0.2006 (0.3555)/0.2214 (0.3514)	0.1973 (0.3433)/0.2359 (0.4024)	0.2243 (0.3342)/0.2399 (0.3289)	0.2266 (0.3559)/0.2499 (0.3766)	0.2260 (0.3859)/0.2551 (0.3958)
Atomic *B*, overall (Å^2^)	45.83	44.07	46.62	31.57	29.95	33.00	32.61	34.60	38.82
Atomic *B*, protein (Å^2^)	45.84	43.97	46.23	31.13	29.55	31.71	32.10	34.14	38.30
Atomic *B*, water (Å^2^)	45.24	40.10	43.71	38.50	35.41	37.36	35.21	38.14	38.65
R.m.s.d., bond lengths (Å)	0.007	0.007	0.008	0.006	0.007	0.012	0.007	0.007	0.012
R.m.s.d., angles (°)	0.78	0.83	0.92	0.71	0.82	1.21	0.86	0.82	1.24
Ramachandran, favoured (%)	98.8	98.8	98.8	100.0	98.8	100.0	99.4	99.4	100.0
Ramachandran, outliers (%)	0.0	0.0	0.0	0.0	0.0	0.0	0.0	0.0	0.0
PDB code	29ik	29il	29im	28ua	28ub	28uc	28ji	28jh	28jg

† CC* for the rotational data sets was calculated according to CC* = [2CC_1/2_/(1 + CC_1/2_)]^1/2^ (Karplus & Diederichs, 2012 ▸).

**Table 2:** Comparison of the compound visibility and model quality with respect to the electron-density maps

	SFX		SSX		Single rotational, cryo	
Compound	DPBA	BXA	DPBA	BXA	DPBA	BXA
*F*_o_(complex) − *F*_o_(apo) peak	10.8σ	11.3σ	8.6σ	7.5σ	13.3σ	18.0σ
*F*_o_(complex) − *F*_o_(apo) integral	2392	2915	2243	2362	2976	7870
Compound RSCC	0.95	0.67	0.96	0.83	0.96	0.9
Compound *B* factors† (Å^2^)	27/38/61	28/61/79	15/25/49	22/73/109	20/28/45	24/51/77
Occupancy‡	0.3	0.3	0.4	0.3	0.4	0.3
PDB code	29il	29im	28ub	28uc	28jh	28jg

† Minimum/median/maximum *B* factor of the compound atoms.

‡ Estimation of the occupancy using extrapolated structure factors from *Xtrapol*8 (mode: ‘difference map maximization’).
