# Supplementary material for: Perspectives for pharmaceutical screening at XFEL sources using the example of Lassa virus endonuclease
Source: Acta Crystallogr D Struct Biol. 2026 Sep 25;82(Pt 10):1181–94. doi: 10.1107/S2059798326009435 (PMC13632748; doi:10.1107/S2059798326009435)
Supplement: Supplementary file 1 [file d-82-01181-sup1.pdf]

STRUCTURAL BIOLOGY

Volume 82 (2026)

Supporting information for article:

Perspectives for pharmaceutical screening at XFEL sources using the example of Lassa virus endonuclease

Sven Falke, Patrick Y. A. Reinke, Daniel Rosenberg, Pontus Fischer, Jan Meyer, Marina Galchenkova, Alexandra Tolstikova, Valerio Mariani, Johanna M. Senst, Phillip Lewe, Susanne Witt, Armin Wagner, Henry N. Chapman, Mark Hunter, Sebastian Günther and Alke Meents

Table S1 Data collection times of the *FosAKP* SFX datasets. Collection and refinement statistics are shown in Table S2. The total gross time is 104.3 min.

	**Apo, DMSO**	**A6**	**A9**	**A12**	**B2**	**G8**	**G12**	***mean***
**Number of patterns**	53985	34454	81935	34400	34240	34240	34395	*43949.9*
**Net time [min]**	13.25	11.32	19.35	11.4	10.3	10.5	11.6	*12.53*
**Gross time [min]**[1]	13.25	19.32	19.37	12.28	10.3	10.5	19.32	*14.9*
**Effective data collection rate [Hz]**[2]	68	51	71	50	55	54	49	*56.86*

[1]This includes extra time required for detector recalibration after overexposure of pixels (“tripping“).

[2]The full X-ray pulse repetition rate at LCLS is 120 Hz.

Table S2 Data collection parameters and refinement statistics of the *FosAKP* SFX data.

F2X entry compound	Apo, DMSO	A6	A9	A12	B2	G8	G12
**space group**	P 2_12_12	P 2_12_12	P 2_12_12	P 2_12_12	P 2_12_12	P 2_12_12	P 2_12_12
**α = β = γ**	90	90	90	90	90	90	90
**a**	71.26	71.1	69.16	71.08	71.17	71	70.6
**b**	45.02	45.1	45.09	45.03	45.05	45.04	44.97
**c**	91.28	91.21	90.71	91.13	91.17	91.15	91
**Number of patterns**	53985	34454	81935	34400	34240	34240	34395
**Number of hits**	25227	16240	26839	14426	17447	17171	10296
**Indexed patterns**	22291	15492	25854	13881	16754	16508	9079
**Indexed crystals**	31029	20398	36896	19005	23038	23017	12314
**Resolution range**	15.46-1.5 (1.55-1.5)	15.34-1.6 (1.66-1.6)	15.22-1.6 (1.66-1.6)	15.34-1.7 (1.76-1.7)	15.38-1.6 (1.66-1.6)	15.33-1.6 (1.66-1.6)	15.37-1.7 (1.76-1.7)
**R_{meas}**	n.d.	n.d.	n.d.	n.d.	n.d.	n.d.	n.d.
**R_{split} (%)**	19.63 (62.01)	28.14 (83.73)	17.64 (77.76)	30.29 (81.15)	29.64 (84.49)	28.61 (88.41)	34.76 (83.22)
**$CC_{1/2}$**	0.9367 (0.594)	0.8717 (0.446)	0.9481 (0.528)	0.8497 (0.379)	0.8495 (0.407)	0.8452 (0.376)	0.7952 (0.457)
**CC***	0.9835 (0.863)	0.9651 (0.785)	0.9866 (0.832)	0.9585 (0.741)	0.9585 (0.761)	0.9571 (0.74)	0.9412 (0.792)
**SNR**	4.29 (0.95)	2.83 (0.45)	3.88 (0.42)	2.92 (0.57)	2.94 (0.47)	2.96 (0.41)	2.66 (0.58)
**Completeness (%)**	100 (100)	100 (100)	100 (100)	100 (100)	100 (100)	100 (100)	100 (100)
**Multiplicity**	367.8 (228.8)	190.1 (118.7)	364.6 (217.6)	180 (117.5)	223.2 (148.2)	224.3 (141.5)	133.2 (88.6)
**Total Measurements**	17580160	7504251	13927309	5925500	8811044	8830516	4347773
**Refinement**							
**Unique Reflections**	47803 (4713)	39485 (3894)	38200 (3759)	32924 (3222)	39472 (3905)	39362 (3897)	32632 (3205)
**WilsonB refinement**	27.38	33.3	35.69	33.78	33.37	34.31	33.15
**Refinement resolution range**	15.38-1.5	15.35-1.6	15.12-1.6	15.44-1.7	15.37-1.6	15.35-1.6	15.44-1.7
**R_{work}**	0.1498	0.1746	0.1485	0.1786	0.1874	0.1764	0.1709
**R_{free}**	0.1801	0.2153	0.223	0.2175	0.2224	0.2179	0.2435
**atomic B overall**	28.52	35.41	36.88	34.82	34.58	36.62	37.08
**atomic B protein**	27	34.3	35.87	33.76	33.46	35.54	36.21
**atomic B water**	43.44	48.75	48.97	47.53	48.01	49.15	47.93
**RMS bond (Å)**	0.006	0.006	0.006	0.007	0.005	0.004	0.004
**RMS angles (°)**	0.927	0.881	0.845	0.775	0.779	0.71	0.718
**Ramachandran, favored (%)**	99.63	99.63	99.26	99.63	99.63	99.26	99.26
**Ramachandran, outliers (%)**	0	0	0	0	0	0	0

Numbers in parentheses refer to the outer shell.

Table S3 Data collection parameters and refinement statistics of *FosAKP* SSX datasets from automatic refinement pipeline (P09/HiPhaX, PETRA III), adopted from (Günther *et al.*, 2025).

F2X entry compound	Apo, DMSO	A6	A9	A12	B2	G8	G12
space group	P 2_12_12	P 2_12_12	P 2_12_12	P 2_12_12	P 2_12_12	P 2_12_12	P 2_12_12
α = β = γ	90	90	90	90	90	90	90
a	71.69	71.62	68.92	71.84	71.43	69.42	71.67
b	91.64	91.63	90.67	91.69	91.63	91.03	91.76
c	45.22	45.16	45.26	45.26	45.28	45.28	45.31
Number of patterns	33439	31892	31892	27740	31892	33265	33439
Number of hits	23756	23669	22800	19567	27850	22118	22977
Indexed patterns	19098	18654	19347	17836	7216	12787	20163
Indexed crystals	24837	25959	26395	24381	8914	16716	27966
Resolution	91.64-1.4 (1.45-1.4)	91.63-1.3 (1.35-1.3)	90.67-1.3 (1.35-1.3)	91.65-1.4 (1.45-1.4)	91.63-1.4 (1.45-1.4)	91.03-1.4 (1.45-1.4)	91.76-1.4 (1.45-1.4)
R_{meas}	n.d.	n.d.	n.d.	n.d.	n.d.	n.d.	n.d.
R_{split} (%)	15.31 (79.84)	15.6 (222.98)	11.97 (127.02)	10.53 (120.12)	26.73 (152.38)	11.23 (260.61)	14.39 (94.33)
$CC_{1/2}$	0.9628 (0.391)	0.965 (0.158)	0.9584 (0.223)	0.989 (0.346)	0.8788 (0.037)	0.9879 (0.128)	0.9695 (0.4)
CC*	0.9905 (0.75)	0.9911 (0.523)	0.9893 (0.604)	0.9972 (0.717)	0.9672 (0.266)	0.9969 (0.476)	0.9922 (0.756)
SNR, overall	5.94 (1.25)	4.91 (0.47)	6.61 (0.91)	6.75 (0.84)	3.50 (0.63)	6.64 (0.74)	6.08 (1.09)
Completeness (%)	100 (100)	99.91 (99.37)	99.91 (99.21)	99.96 (99.63)	99.41 (96.86)	97.22 (77.18)	100 (99.97)
Multiplicity	97.2 (29.6)	86.7 (13)	122.4 (16.3)	127.6 (20.7)	33.8 (12.3)	127.8 (4.9)	102.3 (25.8)
Total Measurements	5782632	6414455	8615031	7607727	1992820	7125788	6104461
**Refinement**							
Unique Reflections	59520 (5844)	73991 (7260)	70384 (6887)	59629 (5846)	59038 (5654)	55772 (4373)	59690 (5855)
WilsonB refinement	16.43	16.54	14.13	16.05	16.57	14.41	16.18
Refinement resolution range	45.82-1.4	45.81-1.3	54.87-1.3	45.85-1.4	56.34-1.4	45.51-1.4	45.88-1.4
R_{work}	0.154	0.1555	0.1723	0.1548	0.1922	0.1532	0.1496
R_{free}	0.1746	0.1787	0.2026	0.1771	0.2212	0.191	0.1745
atomic B overall	20.85	22.56	19.48	20.85	21.38	20.64	21.4
atomic B protein	19.34	21.03	17.88	19.42	19.86	19.3	19.8
atomic B water	35.31	37.1	33.15	34.15	35.58	34.23	36.29
RMS bond (Å)	0.005	0.009	0.007	0.006	0.009	0.009	0.004
RMS angles (°)	0.906	1.005	0.945	0.898	0.85	0.985	0.819
Ramachandran, favored (%)	100	100	99.63	100	100	99.63	100
Ramachandran, outliers (%)	0	0	0	0	0	0	0

Numbers in parentheses refer to the outer shell.

Table S4 Data collection parameters and refinement statistics of *FosAKP* single crystal rotational MX datasets collected at 100 K from automatic refinement pipeline (P11, PETRAIII), adopted from (Günther *et al.*, 2025).

F2X entry compound	Apo, DMSO	A6	A9	A12	B2	G8	G12
space group	$P\ 2_12_12$	$P\ 2_12_12$	$P\ 2_12_12$	$P\ 2_12_12$	$P\ 2_12_12$	$P\ 2_12_12$	$P\ 2_12_12$
α = β = γ	90	90	90	90	90	90	90
a	68.069	68.171	67.942	68.069	68.057	68.068	67.789
b	89.934	89.756	89.797	89.816	89.706	89.838	89.623
c	44.904	44.959	44.924	44.928	44.96	44.943	44.89
Resolution, overall	54.276-1.163 (1.183-1.163)	54.288-1.163 (1.183-1.163)	54.181-1.11 (1.129-1.11)	54.25-1.273 (1.295-1.273)	54.219-1.176 (1.196-1.176)	54.254-1.11 (1.129-1.11)	54.065-1.236 (1.258-1.236)
R_{meas}	0.067 (1.222)	0.07 (1.182)	0.052 (0.849	0.122 (1.986)	0.11 (1.706)	0.054 (0.869)	0.087 (1.394)
R_{split} (%)	n.d.	n.d.	n.d.	n.d.	n.d.	n.d.	n.d.
$CC_{1/2}$	1 (0.653)	1 (0.601)	1 (0.588)	0.999 (0.573)	0.999 (0.523)	1 (0.633)	0.999 (0.679)
CC*	1 (0.889)	1 (0.866)	1 (0.861)	1 (0.854)	1 (0.829)	1 (0.88)	1 (0.899)
SNR	18.1 (1.2)	17.1 (1.2)	23.7 (1.3)	11.5 (1.1)	13.4 (1.1)	22.4 (1.2)	14.3 (1.2)
Completeness (%)	89.7 (59.4)	95 (60.9)	85 (25.5)	99.9 (98.9)	88.3 (54.6)	88.7 (25.7)	95.2 (97.8)
Multiplicity	11.7 (5.2)	11.8 (5)	11.5 (2.9)	13.1 (10.5)	12.4 (6.7)	11.1 (2.7)	12.6 (7.2)
Total Measurements	1002421	1069944	1063658	953630	1008501	1081375	946345
**Refinement**							
Unique Reflections	85527 (2807)	90699 (2885)	92775 (1373)	72797 (3543)	81277 (2488)	97140 (1404)	75124 (3786)
WilsonB refinement	12.69	12.07	10.96	13.54	11.11	11.28	13.31
Refinement resolution range	54.28-1.16	54.29-1.16	54.18-1.11	54.25-1.27	54.22-1.18	54.25-1.11	54.07-1.24
R_{work}	0.1411	0.1393	0.141	0.1449	0.148	0.1346	0.1432
R_{free}	0.1634	0.156	0.1597	0.1667	0.1686	0.146	0.1646
atomic B overall	17.07	16.39	15.19	17.58	15.77	15.23	17.3
atomic B protein	14.63	14.08	12.57	15.25	13.31	12.82	14.67
atomic B water	28.55	27.39	26.44	29.2	27.65	26.33	30.01
RMS bond (Å)	0.016	0.006	0.011	0.007	0.005	0.008	0.009
RMS angles (°)	1.472	1.008	1.22	1.017	0.898	1.078	1.095
Ramachandran, favored (%)	98.89	98.89	98.15	99.63	99.26	98.89	99.26
Ramachandran, outliers (%)	0	0	0	0	0	0	0

Numbers in parentheses refer to the outer shell. CC* was calculated according to $CC^*=\sqrt{\frac{2CC_{1/2}}{1+CC_{1/2}}}$.
